# Association of excessive screen time exposure with ocular changes leading to astigmatism in children

**DOI:** 10.1371/journal.pone.0317961

**Published:** 2025-04-01

**Authors:** Mutahir Shah, Satheesh Babu Natarajan, Nafees Ahmad

**Affiliations:** 1 Department of Applied Sciences, Sub Division Health Sciences, Lincoln University College, Pata Lang Jaya, Selangor, Malaysia; 2 Institute of Biomedical and Genetic Engineering, Islamabad, Pakistan.; Instituto Superior de educação e Ciencias, ISEC Lisboas, PORTUGAL

## Abstract

**Purpose:**

The purpose of this study was to find the relationship between excessive screen time exposure and the development of astigmatism in children.

**Methods:**

A cross-sectional correlational study was conducted at tertiary care hospital, Islamabad between June 2023 to May 2024. In total, 431 patients were included in this study after informed written consent. Screen time exposure was assessed through smartphone usage history or parental reports of daily use of smart gadget(s). Tear break-up time was measured using a fluorescein strip, cobalt blue filter on a slit-lamp biomicroscope and a stopwatch. Data was analyzed using univariate and multivariate statistical tests including Spearman’s correlation and regression analyses.

**Results:**

The study included 431 children (mean age: 6.70 ±  1.80 years; 55% male, 45% female). Mean screen time was 4.54 ±  1.52 hours/day. A positive correlation between screen time and the magnitude of astigmatism was observed (r = 0.33, p < 0.001). Regression analysis showed a significant relationship among screen time and astigmatism (B = 0.177, CI: 0.80-0.25). Tear break- up time (TBUT) showed a significant negative correlation with screen time (r = -0.167, p < 0.001), and reduced TBUT was linked to a higher risk of inflammatory conjunctivitis and lid thickening (B = -0.431, CI: -0.12 to -0.49, p < 0.001). The results highlighted that inflammatory conjunctivitis/lid thickness have three times greater risk of developing high astigmatism (OR =  3.31, p-value <  0.001, CI =  1.91 to 5.73) while the risk of moderate astigmatism in such cases was two times higher (2.12, p-value =  0.004, CI = 1.26-3.56). However, the effect of lid thickness on astigmatism when combining with screen time has a little effect that is not significant (p-value = 0.053). Thus, excessive screen time is an independent risk factor of causing astigmatism in children (p < 0.001). The Wilcoxon Signed Ranks Test demonstrated significant improvement in visual acuity after correction (p < 0.001).

**Conclusion:**

Excessive screen time in children is significantly associated with astigmatism, tear film instability, inflammatory ocular conditions, including conjunctivitis and lid thickening. These findings suggest the need for preventive strategies, such as reducing screen time and encouraging regular eye examinations, to protect children’s ocular health.

## Introduction

Emerging technologies, such as smart screen gadgets and interactive screen media, have become an integral part of youth and children’s daily life. These technologies are now widespread and significantly influence our daily lives, including the habits of youngsters and children [1,2]. Today’s children are genuinely “digital natives” in a constantly evolving digital world. The average age at which children begin interacting with media has decreased from 4 years to 4 months since 1970 [3]. While digital devices offer educational and recreational benefits, excessive screen time exposure has raised significant concerns regarding ocular and general health. The potential detrimental effects on children’s eye health and development have prompted worries about the alarming increase in screen usage, as evidenced by multiple studies [1,2,4]. Most pediatric associations worldwide recommended that children should limit their recreational screen exposure to less than 2 hours per day in response to these concerns [3,5].

Exposure to excessive screen time during early life is linked with refractive errors. Uncorrected refractive errors are responsible for 43% of global visual impairment [6,7]. They were further classified into myopia, hypermetropia and astigmatism. Myopia is the commonest refractive error that required correction in early age. Its causes, risk factors and management options have been extensively reported [8]. While astigmatism is relatively less studied among refractive errors. It became more prevalent worldwide in recent years, raising important clinical and public health concerns [9]. If left untreated, astigmatism significantly reduces visual acuity, negatively affects child’s visual development, and may result in amblyopia [10,11].

Apart from that, studies showed that prolonged exposure to screens in children is associated with abnormal ocular surface and dry eyes [12–14]. Excessive screen use is thought to be a contributing factor to dry eye diseases (DED), as it alters blink patterns, leading to an abnormal ocular surface. Decreased spontaneous blinking and incomplete eyelid closure during prolonged screen time affect tear film stability, leading to increased aqueous evaporation, altered osmolarity, and the buildup of inflammatory mediators [15]. These inflammatory mediators are proposed to be associated with an ocular inflammatory response. This ocular inflammatory response is the key to inflammatory conjunctivitis that is proposed to cause lid thickening. This mechanism may cause mechanical reshaping of the cornea. Previous studies had shown that, in children and teenagers, factors such as extraocular muscle tone, excessive screen time, eyelid pressure, heredity, visual input, and environmental pollution may play a role in the pathogenesis of astigmatism, although the exact pathogenesis remains unclear [9,10,16,17]. Thus, there is a gap in the current literature regarding direct or indirect association of screen time with the development of astigmatism. Identifying modifiable risk factors that may contribute to the development of astigmatism during the critical period is therefore essential.

The aim of this study was to investigate the relationship between excessive screen time exposure and the development of astigmatism in the pediatric population. Furthermore, the study highlighted the effect of thickened upper lid due to dry eyes on development of astigmatism.

## Methodology

This cross-sectional correlational study was conducted in the department of ophthalmology at tertiary care hospital, Islamabad. Ethical approval was granted by the hospital’s ethical review committee ref no:XXX-HI-PUB-ERC/Mar23/24. All steps were followed as per the guidelines of the Declaration of Helsinki. Informed written consent was obtained from the parents/guardians. Additional information regarding the ethical, cultural, and scientific considerations specific to inclusivity in global research is included in the supporting information (Inclusivity-in-global-research-questionnaire). In this study, 431 individuals aged 3 to 11 years were included through a consecutive sampling technique (non-probability sampling). The sample size was calculated using WHO calculator with a 95% CI and considering pediatric astigmatism prevalence of 14.9% (0.149) [1]. The study was conducted from June 2023 to May 2024. Participants with headaches, squint, unhabitual blinking, meridional amblyopia,astigmatism ≥  -0.75, and a history of excessive gadget use, i.e., smartphones, tab and laptop ( ≥ 2 hours/day for ≥  6 months) were included. Exclusion criteria included children with retinal dystrophies and diseases, trauma, ectasias, keratoconus (following Rabinowitz criteria), Ehlers-Danlos syndrome, Down syndrome, Marfan syndrome, vernal keratoconjunctivitis, atopy, history of contact lens use, history of squint surgery, maternal age > 35 years [2], birth weight < 1500g [3], gestational period < 37 weeks [2] and history of ocular surgeries or any systemic illness. Exposure to TV screens was excluded from the study because recent research predominantly associates it with myopia rather than astigmatism [4]. Screen time was evaluated using smartphone usage history, supported by parental reports for children with dedicated gadgets. For non-dedicated gadgets, parental reports on their children’s usage were utilized (S1 Fig. The figure highlights screen time usage among children with dedicated devices).

Visual acuity (uncorrected and corrected) was measured using a log MAR visual acuity chart held at a distance of 4 meters. Refraction was performed with an autorefractometer and retinoscopy using (Nidek ARK-1, Japan) and retinoscope (NIETZ, Japan). Cycloplegic refraction was performed after instilling 1% cyclopentolate drops three times, with a duration of 10 minutes between each instillation, and the child was re-examined after 60 minutes [18]. Corneal curvature was measured using an OCT-based corneal topography instrument (OPTOPOLE Revo NX-130, Poland).

Tear break-up time was documented using fluorescein strip instillation, followed by evaluation with a cobalt blue filter on a slit-lamp biomicroscope and a stopwatch. Conjunctival tissue was assessed using a slit-lamp biomicroscope for inflammatory response and papillary reactions. The inflammatory conjunctivitis was supposed to be associated with thickening of the tarsal plat of the conjunctiva [2]. The thickening or swelling of the conjunctival tarsal plate was clinically assessed on slit lamp examination based on conjunctival inflammation and digital assessment for overall lid rigidity and thickness. Blink rate while staring at a smartphone was measured using a video analyzer. Ocular deviation was measured using loose prisms and alternate cover test.

Data were analyzed using SPSS version 26. Descriptive statistics were applied for continuous variables (e.g., age, visual acuity, astigmatism magnitude), while categorical variables were graphically presented. The data did not follow a normal distribution according to the Shapiro-Wilk test criteria and was further confirmed by the normality assessment using histogram plots (S2 Fig. The figure showed the graphical presentation of the histogram plots highlighting the normality of the data). Spearman’s correlation analysis was performed to find the association between the magnitude of astigmatism and screen time. Univariate analysis was conducted among dependent and independent variables along with gender and family history. In multivariable linear regression model assessed the relationship between screen time and astigmatism, TBUT, and conjunctival inflammation. Bonferroni adjustment for multiple comparisons was utilized to establish the p-value for the multivariable analysis which was calculated to be 0.025. Chi-square tests, binary, and multinomial logistic regression analyzed associations between TBUT, astigmatism, lid thickness, and conjunctivitis. Wilcoxon Signed-Rank test was used for visual acuity comparison. A p-value of ≤  0.05 was considered as statistically significant (SPSS data).

## Results

**Fig 1:**
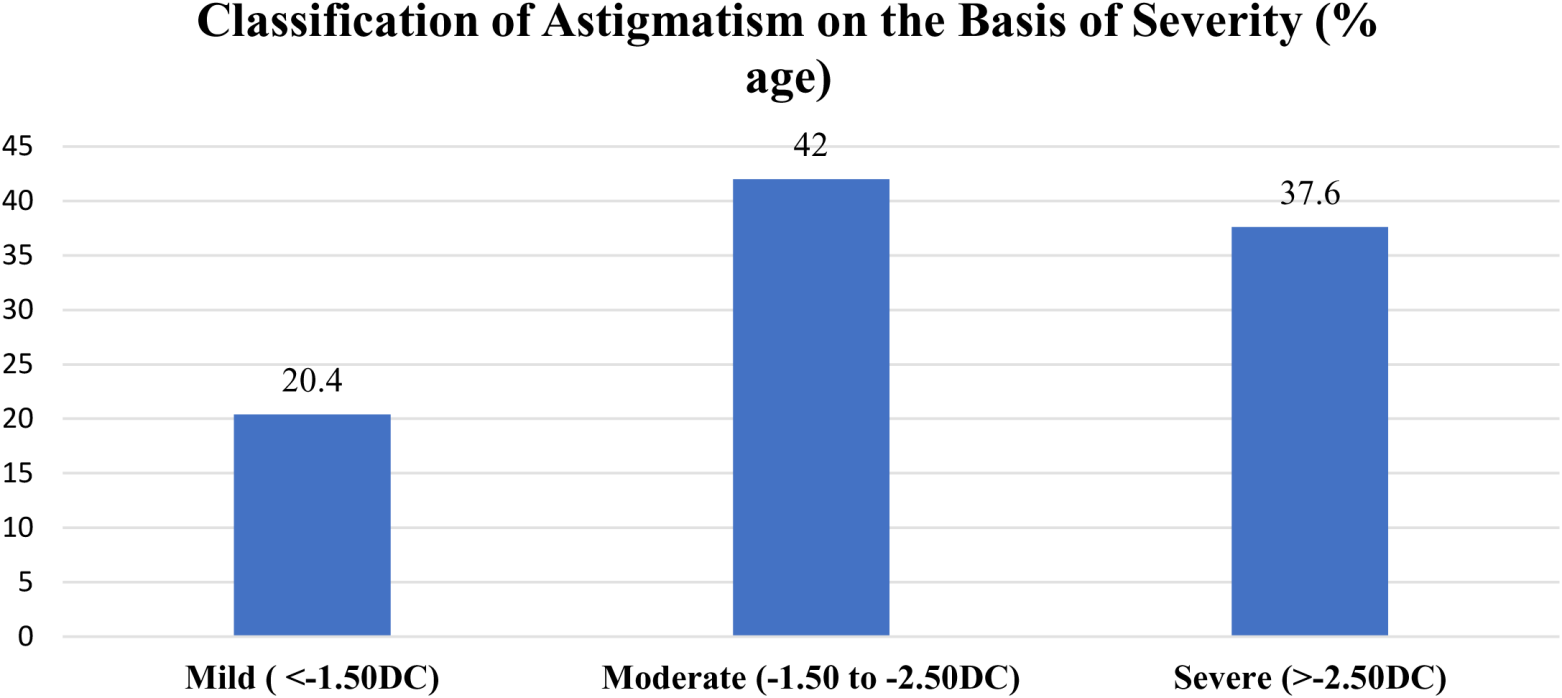
Showed classification and magnitude of astigmatism based on severity. Majority of the cylindrical prescription were lying in moderate and high form (Mild -0.75 to -1.50, Moderate> -1.50 to -2.50, High > -2.50).

In total 431 patients were included in this study. The study population consisted of children with a mean age of 6.70 ±  1.80 years. Both genders were included in the study, with 55% males and 45% females. The feeding patterns of the children were breastfed (69%), followed by bottle-fed (15.5%) and mixed feeding (15.5%). Birth history showed a balanced distribution between vaginal delivery (VD; 51.7%) and caesarean section delivery (CSD; 48.3%). A family history of refractive error (myopia, hyperopia, astigmatism) was positive in 25.5% of the participants. Parents were educated to graduate or postgraduate levels (S3 Fig. This figure showed parents educational level of the study participants. It showed that mostly they were educated to graduate and post graduate level.), with incomes typically falling within tax slabs 3 (2.2-3.1 million per annum) and 4 (3.1-4.2 million per annum) designated by the government of Pakistan (S4 Fig. Presented the status of parental income per annum as per Govt of Pakistan tax slab status, majority were lying in tax slab 3 and 4). Intermittent exotropia was common among the study population. Collinearity for all variables between the right and left eyes was highly significant (p-value <  0.001), so only the results of the right eye were included. The average uncorrected visual acuity was 0.59 ±  0.18 LogMAR (0.511 Log MAR in hormonic means value), which improved with spectacle correction to a mean of 0.18 ± 0.14 LogMAR. The mean screen time exposure was 4.54 ±  1.52 hours per day, and the average outdoor activity duration was 30.6 minutes. The mean total and corneal astigmatism were -2.44 ± 1.15DC and -2.38 ± 0.94DC, respectively (Table 1). Astigmatism severity was categorized into three groups: mild (≥-0.75 to -1.50DC), moderate (>-1.50 to -2.50DC) and high (>-2.50DC). Group 1 consisted of 87 participants, while group 2 had 182 and group 3 had 162 participants (Fig 1). The data also included tear break-up time (10.34 ±  3.19 seconds) and blink rate (10.07 ±  3.03 blinks per minute; Table 1). The mean corneal thickness was 542 ±  32.43µm (480-610). All study participants showed astigmatism “with the rule” and it was mostly corneal in nature. Mixed astigmatism was the most prevalent type of astigmatism (47.8%), followed by compound hyperopic astigmatism (19.3%), simple myopic astigmatism (16.7%), compound myopic astigmatism (11.6%) and the least common was simple hypermetropic astigmatism (4.6%; S5 Fig. Represented the types of Astigmatism on the basis of Foci, it showed that majority of the study participants were diagnosed with Mixed Astigmatism followed by compound hyperopic, Myopic and simple Hyperopic Astigmatism).

**Table 1:** Descriptive analysis of demographic and clinical parameters (n = 431).

Demographics and Clinical Data	Mean ± SD	Minimum	Maximum
Age	6.67 ± 1.90	3	11
Birth Weight	3.01 ± 0.34	2.00	4.20
Gestational Period (weeks)	38.75 ± 1.36	37	42
Visual Acuity Uncorrected (LogMAR)	0.59 ± 0.18	1.20	0.10
Visual Acuity Corrected (LogMAR)	0.18 ± 0.14	0.00	0.50
Corneal Astigmatism (DC)	-2.38 ± 0.94	-0.75	-6.0
Magnitude of Astigmatism (Total, DC)	-2.44 ± 1.15	-0.75	-6.50
Exposure to Screen Time in Hours	4.54 ± 1.52	2.0	13.00
Outdoor Activity in Minutes	30.6 ± 11.34	15.0	120.00
Tear Break Up Time in seconds	10.34 ± 3.19	4	22
Residual Astigmatism (DC)	-0.08 ± 1.35	-2.50	1.75
Near Point of Convergence (cm)	7.41 ± 1.51	0.00	14.00
Magnitude of Deviation (pd)	6.27 ± 11.75	0	50
Blink Per Minute	10.07 ± 3.03	3	22
Corneal Pachymetry	542 ± 32.43	480	610

Symptoms of inflammatory conjunctival tissue were common in approximately 59.80% of participants, correlating with a thick tarsal conjunctival plate. The mean value of tear film stability, as measured by tear break-up time, was 10.80 ±  3.25 seconds (Table 1). The data revealed that 48.5% of children exposed to gadgets were diagnosed with abnormal tear film. The classification of tear break-up time showed mild TBUT in 31.1%, moderate in 16.5%, and severe in 0.9% of cases. The mean blink rate while using screens was 10.12 ±  3.0 blinks per minute, which is less than the average blink rate of 15-25 times per minute. Limited outdoor activities were reported by 87.67% of participants. Meridional amblyopia was found in 54.26% of the study participants.

**Fig 2:**
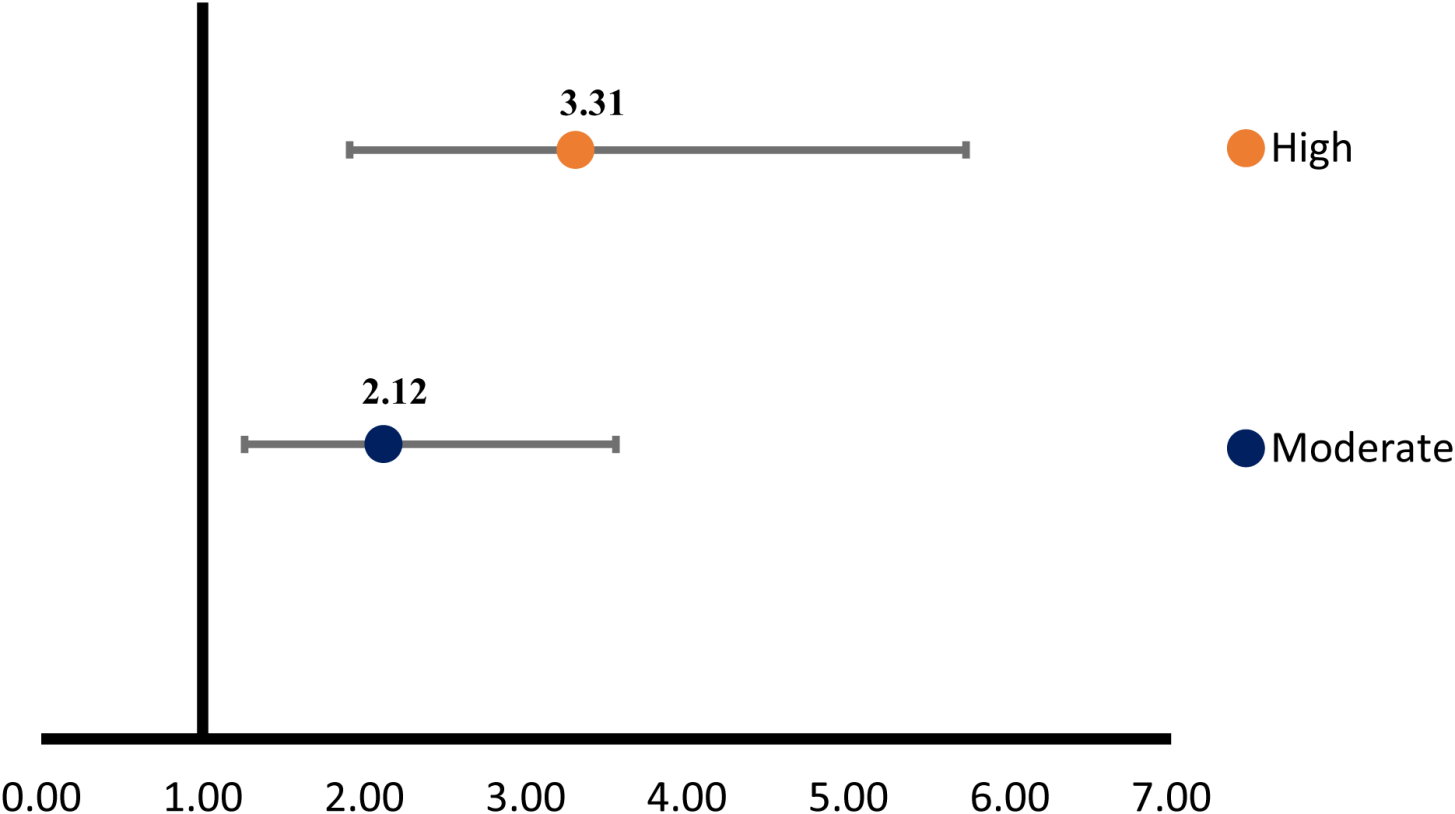
The forest plot representing the results of association between thickening of the upper eye lid due to inflammatory conjunctivitis in patients exposed to excessive screen time and astigmatism grades, i.e., mild, moderate and high. The plot visually displays the strength and significance of the association, with the horizontal line representing the confidence interval and the dots marking the odds ratio. Since the confidence interval does not cross this line, it suggests a statistically significant association. (Ref value 1: Mild Astigmatism -0.75 to -1.50DC).

The univariate analysis showed significant relationship among screen time exposure (independent variable), total astigmatism, and TBUT, while the results were insignificant for family history and gender analysis (Tables 2 and 3). The magnitude of total and corneal astigmatism showed a positive correlation with screen time (r = 0.33, p-value <  0.001; Table 4 ). This indicates that increasing screen time is associated with an increasing magnitude of astigmatism. The association of screen time and magnitude of astigmatism was further explored using regression analysis that showed a significant relationship (B = 0.177, p < 0.001; Table 3). It revealed that as the screen time increases, the magnitude of astigmatism also increases. The screen time and TBUT showed a significant negative correlation (r =  -0.167, p-value <  0.001). However, the association between TBUT and inflammatory conjunctival tissue/lid thickness was significant (χ² =  166.787; p <  0.001; Table 4), the regression analysis highlighted that unstable tear film (B =  -0.431, p <  0.001; Table 3) is a risk factor for causing inflammatory conjunctivitis and lid thickening. The risk of inflammatory conjunctival tissue and lid thickness increases by 35% for each second of decrease in TBUT. Patients with inflammatory conjunctivitis/lid thickness had significantly higher odds of developing high astigmatism (OR =  3.31, p-value <  0.001, CI =  1.91 to 5.73) and moderate astigmatism (OR =  2.12, p-value =  0.004, CI =  1.26 to 3.56) Fig 2, Table 5). However, the effect of lid thickness on astigmatism when combining with screen time has a little effect that is not significant (p-value = 0.053). Thus, screen time is an independent risk factor of causing astigmatism in children (p < 0.001). The Wilcoxon Signed Ranks Test indicated a statistically significant improvement in visual acuity after correction. The visual acuity was significantly better in patients with correction (Z = 17.992; p-value <  0.001)

**Table 2:** Univariate analysis among excessive screen time exposure and dependent variables.

Screen time Exposure (Constant)	B	t value	p-Value	Conf. Interval
Total Astigmatism	0.177	3.732	<0.001	0.080-0.254
TBUT	-0.154	-3.233	<0.001	-0.50- -0.12
Gender	0.078	1.625	0.105	-0.050-0.524
Family History	0.031	0.648	0.518	-0.220-0.436

**Table 3:** Effect of excessive screen exposure on total astigmatism, corneal astigmatism and TBUT.

Screen time Exposure (Constant)	B	t value	p-Value	Conf. Interval
**Total Astigmatism and Corneal Astigmatism**	0.177	3.732	<0.001	0.080 to 0.254
**TBUT**	-0.154	-3.233	<0.001	-0.50 to -0.12

* Bonferroni Correction: 0.025.

**Table 4:** The Spearman correlation analysis revealed a statistically significant negative correlation among exposure to screen time and TBUT while positive correlation with total astigmatism.

Exposure to Screen time in Hours (Constant)	Correlation Coefficient (rho)	95% Confidence Interval	p-Value
Total Astigmatism	0.327	1.0 - 0.24	<0.001
Tear Break Up Time	-0.167	-0.074 - -0.258	

**Table 5:** The results highlighted the odds ratio of inflammatory conjunctivitis related lid thickness and corneal astigmatism in patients exposed to excessive screen time.

Sr. No	Astigmatism	Odds ratio	95% CI (Lower – Upper)
1	Low (-0.75 to -1.50)	Ref	
2	Moderate (>-1.50 to -2.50DC)	2.12	1.26 – 3.56
3	High (>-2.50DC)	3.31	1.91 – 5.73

## Discussion

This study investigated the effect of excessive screen time exposure on visual and ocular health, specifically focusing on the relationships between excessive screen time, astigmatism, tear film stability (TBUT) and inflammatory conjunctivitis. With the rule astigmatism was observed in all children, and the most common type was mixed astigmatism. Moreover, there were factors associated with forceful blinking and lid thickening. Exotropia was more frequently observed in patients with uncorrected astigmatism

The risk of developing astigmatism potentially due to screen time exposure based on gender have contradictory reports and seems to vary in different populations. In this study we found that males were more prone to the effects of screen time that leads to astigmatism compared to female children. These findings are supported by studies from other populations [11,19]. Conversely, some studies have indicated that the prevalence of astigmatism is significantly higher among female students compared to their male counterparts while others have found no correlation between astigmatism and gender [20,21]. A major outcome of this study was the effect of excessive screen time on astigmatism. There are multiple factors associated with astigmatism, including environmental conditions, maternal/paternal smoking (active or passive), genetics, lifestyle, birth history, and feeding patterns. This study revealed that only 2.2% of the children were exposed to smoke during early childhood, suggesting no significant association between astigmatism and parental smoking. Also, most participants were breastfed, as opposed to those who were bottle-fed or had mixed feeding patterns. The findings further indicated that, unlike previous studies, neither bottle feeding, nor c-sections had a significant impact on the development of astigmatism. [10,11,22,23].

A previously published study reported 33.1% prevalence of parental refractive error, while others found that children with a positive family history of astigmatism were more likely to develop astigmatism [6,8]. In comparison, this study observed a similar prevalence of parental refractive error at 25.5%, aligning with previously published data. These findings underscore the genetic predisposition to refraractive error specifically astigmatism, as demonstrated in prior research [8]. Moreover, the high education levels and annual incomes of the parents in current study may contribute to enhanced access to healthcare and preventive measures. However, this accessibility may also increase risks, as excessive exposure to electronic devices among children might cause ocular discomfort and the onset of astigmatism.

Excessive screen time is considered a potential risk factor for developing astigmatism, either directly or indirectly. The study highlighted that there is a positive correlation among excessive screen time exposure and astigmatism (p < 0.001). The higher exposure to screen time is associated with a greater magnitude of astigmatism. A significant positive relationship between the amount of screen time in hours and the severity of astigmatism was found (p ≤ 0.001). This suggested that for each additional hour of screen time, the magnitude of astigmatism will increase by 0.166 DC (p < 0.001). Excessive exposure to screens has been identified as a significant factor influencing vision development [24]. This may be linked to the impact on lens development, the low tensile strength of the cornea, pressure of upper lid on cornea in lower gaze and alterations in corneal shape due to prolonged exposure to smart screens [11,25]. This notion is supported by a study conducted in the Longhua district of Shenzhen among preschool children, where a significant association was found between early screen exposure and an elevated risk of astigmatism [10]. The risk of developing astigmatism was positively correlated with duration of screen exposure. Similar findings are also reported in other studies, further validating our results [26,27]. This study aligns with the previously published studies, showing a marked increase in astigmatism prevalence when daily screen time exceeds 2 hours, highlighting the importance of managing screen time to mitigate its impact on ocular health.

The results of excessive screen time exposure and TBUT revealed a negative correlation (p < 0.001; Table 4). This suggests that excessive screen time is associated with reduction in tear film stability. Specifically, for each additional hour of screen time, TBUT decreases by approximately 0.306 seconds. This suggests that higher screen time is associated with faster tear film breakup, potentially indicating a higher risk of dry eye symptoms or discomfort. Symptomatic DEDs associated with excessive screen time are linked to incomplete or partial blinking, which may lead to altered osmolarity and the buildup of inflammatory mediators. These inflammatory mediators are responsible for causing inflammatory conjunctivitis [28]. This study revealed a significant association between TBUT and inflammatory conjunctivitis/lid thickness (χ² =  166.787, p <  0.001). The results of TBUT and inflammatory conjunctivitis was significant (p < 0.001) supporting strong evidence of association between these variables that leads to thickening of the lid tarsal plate (Table 5). To explore the role of inflammatory conjunctivitis/lid thickness in the development of astigmatism, odds ratios were calculated (Fig 2). It showed that individuals with inflammatory conjunctivitis/lid thickening were significantly more likely to develop astigmatism, supporting the theory of mechanical reshaping of the cornea. However, the multinomial regression analysis of astigmatism and thickened tarsal conjunctival plate in relation to screen time revealed that, although lid thickness is associated with astigmatism, its impact is minor. In contrast, screen time emerged as an independent and significant risk factor for astigmatism development. The study also showed that not every child has a thicker lid due to inflammatory conjunctivitis. Prolonged lower gaze while staring at a screen itself has the potential to reshape the cornea, as its tensile strength is less in young individuals. The physiological link between screen exposure and astigmatism remains unclear, though several hypotheses exist. One possibility is that prolonged screen time at close distances may lead to excessive accommodation, which overworks the ciliary muscles in children’s eyes and potentially disrupts the natural development of the crystalline lens, altering its curvature [24]. Another hypothesis suggests that close-up screen viewing could change the shape of children’s corneas due to variations in the palpebral aperture and eye movements during these tasks or due to mechanical interactions between the cornea and eyelids [29]. Additionally, it has been proposed that looking down at a screen may increase pressure from the eyelids on the cornea, potentially leading to steepening of the vertical corneal meridian, causing with-the-rule astigmatism [25,30] Other findings of the study was presence of exotropia mostly intermittent that were common among children exposed to screen time and astigmatism. Astigmatic blur is one of the reasons that can contribute to the disruption of binocular vision, particularly in individuals with weak fusional control, potentially leading to the development of exotropia. A recently published studies by Çakır B et al. and Shah M et al. revealed that intermittent exotropia is more common in anisoastigmatic children [16,31].

### Limitation of the study

This cross-sectional study highlights the association and risk factors of excessive screen time exposure with changes in ocular dimensions, particularly astigmatism, in children. While we have made every effort to minimize biases related to screen time exposure, it remains a limitation of the study.

## Conclusion

The current study highlighted the significant impact of excessive screen time on various aspects of visual and ocular health in children. The important aspects were the development of astigmatism, tear film instability, and associated conditions such as inflammatory conjunctivitis and exotropia. The study also revealed the adverse effect of screen time on TBUT, contributing to digital eye strain and dry eye symptoms. Furthermore, the research suggests that inflammatory conjunctivitis and lid thickening, exacerbated by screen time, may play a role in the mechanical reshaping of the cornea, leading to astigmatism. These results emphasize the need for preventive measures, such as limiting screen time and promoting regular eye examinations, to mitigate the adverse effects of smart screens on children’s ocular health.

## Supporting information

S1 Fig The figure highlights screen time usage among children with dedicated devices. (PDF)

S2 Fig The figure showed the graphical presentation of the histogram plots highlighting the normality of the data. (PDF)

S3 Fig This figure showed parents educational level of the study participants. It showed that mostly they were educated to graduate or post graduate level.
(PDF)

S4 Fig Presented the status of parental income status per annum as per Govt of Pakistan Tax Slab status, Majority were lying in tab slab 3 and 4. (PDF)

S5 Fig Represented the type of Astigmatism on the basis of Foci, it showed that majority of the study participants were diagnosed with Mixed Astigmatism followed by Myopic and Hypermetropic Astigmatism. (PDF)

S1 File Inclusivity in global research questionnaire. (DOCX)

S2 File SPSS data. File of Association of Escessive Screen time exposure in children.
(SAV)

## References

[1] Radesky JS, Christakis DA. Increased screen time: implications for early childhood development and behavior. Pediatr Clin North Am. 2016;63(5):827–39. doi: 10.1016/j.pcl.2016.06.006 27565361

[2] Guo Y-F, Liao M-Q, Cai W-L, Yu X-X, Li S-N, Ke X-Y, et al. Physical activity, screen exposure and sleep among students during the pandemic of COVID-19. Sci Rep. 2021;11(1):8529. doi: 10.1038/s41598-021-88071-4 33879822 PMC8058040

[3] Strasburger VC, Hogan MJ. Children, adolescents, and the media. Pediatrics. 2013;132(5):958–61. doi: 10.1542/peds.2013-2656 28448255

[4] Madigan S, Browne D, Racine N, Mori C, Tough S. Association between screen time and children’s performance on a developmental screening test. JAMA Pediatr. 2019;173(3):244–50. doi: 10.1001/jamapediatrics.2018.5056 30688984 PMC6439882

[5] Hale L, Guan S. Screen time and sleep among school-aged children and adolescents: a systematic literature review. Sleep Med Rev. 2015;21:50–8. doi: 10.1016/j.smrv.2014.07.007 25193149 PMC4437561

[6] Hashemi H, Asharlous A, Khabazkhoob M, Yekta A, Emamian MH, Fotouhi A. The profile of astigmatism in 6-12-year-old children in Iran. J Optom. 2021;14(1):58–68. doi: 10.1016/j.optom.2020.03.004 32345571 PMC7752993

[7] Hashemi H, Fotouhi A, Yekta A, Pakzad R, Ostadimoghaddam H, Khabazkhoob M. Global and regional estimates of prevalence of refractive errors: systematic review and meta-analysis. J Curr Ophthalmol. 2017;30(1):3–22. doi: 10.1016/j.joco.2017.08.009 29564404 PMC5859285

[8] Harb EN, Wildsoet CF. Origins of refractive errors: environmental and genetic factors. Annu Rev Vis Sci. 2019;5 47–72. doi: 10.1146/annurev-vision-091718-015027 31525141 PMC11827892

[9] Yang Z, Lu Z, Shen Y, Chu T, Pan X, Wang C, et al. Prevalence of and factors associated with astigmatism in preschool children in Wuxi City, China. BMC Ophthalmol. 2022;22(1):146. doi: 10.1186/s12886-022-02358-2 PMC8976382 35365119

[10] Huang L, Yang G-Y, Schmid KL, Chen J-Y, Li C-G, He G-H, et al. Screen exposure during early life and the increased risk of astigmatism among preschool children: findings from Longhua child cohort study. Int J Environ Res Public Health. 2020;17(7):2216. doi: 10.3390/ijerph17072216 32224959 PMC7177845

[11] Wang Y, Mu J, Yang Y, Li X, Qin H, Mulati B, et al. Prevalence and risk factors for astigmatism in 7 to 19-year-old students in Xinjiang, China: a cross-sectional study. BMC Ophthalmol. 2024;24(1):116. doi: 10.1186/s12886-024-03382-0 38481203 PMC10935971

[12] Muntz A, Turnbull PR, Kim AD, Gokul A, Wong D, Tsay TS-W, et al. Extended screen time and dry eye in youth. Cont Lens Anterior Eye. 2022;45(5):101541. doi: 10.1016/j.clae.2021.101541 34840070

[13] Kocamiş Ö, Temel E, Aşikgarip N, Örnek K. Electronic device screen time and meibomian gland morphology in children. J Ophthalmic Vis Res. 2021;16(4):531–7. doi: 10.18502/jovr.v16i4.9741 34840674 PMC8593533

[14] Alnahdi W, Hadrawi M, Danish E, Alghamdi A, Taher N, Alfaraidi AT, et al. Relationship between screen time and dry eye symptoms during the COVID-19 pandemic in the pediatric population of the western region of Saudi Arabia. Cureus. 2022;14(11):e31015. doi: 10.7759/cureus.31015 36475117 PMC9717719

[15] Bron AJ, de Paiva CS, Chauhan SK, Bonini S, Gabison EE, Jain S, et al. TFOS DEWS II pathophysiology report. Ocul Surf. 2017;15(3):438–510. doi: 10.1016/j.jtos.2017.05.011 28736340

[16] Çakır B, Aksoy NÖ, Özmen S, Bursalı Ö. The effect of amblyopia on clinical outcomes of children with astigmatism. Ther Adv Ophthalmol. 2021;13:25158414211040898. doi: 10.1177/25158414211040898 34541452 PMC8442477

[17] McKean-Cowdin R, Varma R, Cotter SA, Tarczy-Hornoch K, Borchert MS, Lin JH, et al.; Multi-Ethnic Pediatric Eye Disease Study and the Baltimore Pediatric Eye Disease Study Groups. Risk factors for astigmatism in preschool children: the multi-ethnic pediatric eye disease and Baltimore pediatric eye disease studies. Ophthalmology. 2011;118(10):1974–81. doi: 10.1016/j.ophtha.2011.06.031 21856010 PMC3186875

[18] Al-Thawabieh W, Al-Omari R, Abu-Hassan DW, Abuawwad MT, Al-Awadhi A, Abu Serhan H. Tropicamide versus cyclopentolate for cycloplegic refraction in pediatric patients with brown irides: a randomized clinical trial. Am J Ophthalmol. 2024;257 218–26. doi: 10.1016/j.ajo.2023.09.022 37797867

[19] Wang J, Cheng QE, Fu X, Zhang R, Meng J, Gu F, et al. Astigmatism in school students of eastern China: prevalence, type, severity and associated risk factors. BMC Ophthalmol. 2020;20(1):155. doi: 10.1186/s12886-020-01425-w 32306963 PMC7168812

[20] He M, Zeng J, Liu Y, Xu J, Pokharel GP, Ellwein LB. Refractive error and visual impairment in urban children in southern china. Invest Ophthalmol Vis Sci. 2004;45(3):793–9. doi: 10.1167/iovs.03-1051 14985292

[21] Namba H, Sugano A, Murakami T, Utsunomiya H, Nishitsuka K, Ishizawa K, et al. Age-related changes in astigmatism and potential causes. Cornea. 2020;39(1):S34–8. doi: 10.1097/ico.0000000000002507 33038156

[22] Balouch MS, Shahbaz M, Balouch MM, Balouch MS, Abbasi MU. The association between refractive errors and breastfeeding in Pakistani children: a case-control study. Cureus. 2022;14(8):e28311. doi: 10.7759/cureus.28311 36034062 PMC9398200

[23] Zhang J, Wu Y, Sharma B, Gupta R, Jawla S, Bullimore MA. Epidemiology and burden of astigmatism: a systematic literature review. Optom Vis Sci. 2023;100(3):218–31. doi: 10.1097/OPX.0000000000001998 36749017 PMC10045990

[24] Agarwal S, Goel D, Sharma A. Evaluation of the factors which contribute to the ocular complaints in computer users. J Clin Diagn Res. 2013;7(2):331–5. doi: 10.7860/JCDR/2013/5150.2760 23543722 PMC3592304

[25] Shah M, Babu Natarajan S, Ahmad N. Meridional amblyopia and spectacle correction: a prospective interventional study of children aged 4–11 years. Cureus. 2024. doi: 10.7759/cureus.67549 PMC11416691 39310641

[26] Islam B, Nasim M, Shah M, Sharif R, Rashid MK. Screen time and refractive errors. Al-Shifa J Ophthalmol. 2020;16(02):79–84.

[27] Shah M, Ullah S. Impact of gadgets on amblyopic therapy and risk of astigmatism development: A prospective case report of a 5 years old. Al-Shifa J Ophthalmol. 2023;19(03):128–30.

[28] Chu L, Wang C, Zhou H. Inflammation mechanism and anti-inflammatory therapy of dry eye. Front Med (Lausanne). 2024;11 1307682. doi: 10.3389/fmed.2024.1307682 38420354 PMC10899709

[29] Collins MJ, Buehren T, Bece A, Voetz SC. Corneal optics after reading, microscopy and computer work. Acta Ophthalmol Scand. 2006;84(2):216–24. doi: 10.1111/j.1600-0420.2005.00547.x 16637840

[30] Read SA, Collins MJ, Carney LG. The diurnal variation of corneal topography and aberrations. Cornea. 2005;24(6):678–87. doi: 10.1097/01.ico.0000154385.43732.6e 16015086

[31] Shah M, Natarajan SB, Ahmad N. Excessive use of electronic gadgets leads to astigmatism and ocular deviation: A prospective case series study. Xi’an Shiyou Daxue Xuebao (ziran kexue ban)/ Journal of Xi’an Shiyou University, Natural Sciences edition. 2024;67(9):268-277. doi: 10.5281/zenodo.13822195

