# Correction: Association of excessive screen time exposure with ocular changes leading to astigmatism in children

**DOI:** 10.1371/journal.pone.0334319

**Published:** 2025-10-10

**Authors:** Mutahir Shah, Satheesh Babu Natarajan, Nafees Ahmad

There are errors in the author affiliations. The correct affiliations are as follows:

Mutahir Shah^1^, Satheesh Babu Natarajan^2^, Nafees Ahmad^3^

**1** Faculty of Applied Sciences, Sub Division Health Sciences, Lincoln University College, Petaling Jaya, Selangor, Malaysia, **2** School of Pharmacy and Traditional Complementary Medicine, Lincoln University College, Petaling Jaya, Selangor, Malaysia, **3** Institute of Biomedical and Genetic Engineering, Islamabad, Pakistan.
