# Supplementary material for: Association of excessive screen time exposure with ocular changes leading to astigmatism in children
Source: PLoS One. 2025 Apr 1;20(4):e0317961. doi: 10.1371/journal.pone.0317961 (PMC11960901; doi:10.1371/journal.pone.0317961)
Supplement: S1 Fig — (PDF) [file pone.0317961.s001.pdf]

Weekly report

11 March - 17 March (Week 11)

4 h 33 m

Daily average screen time

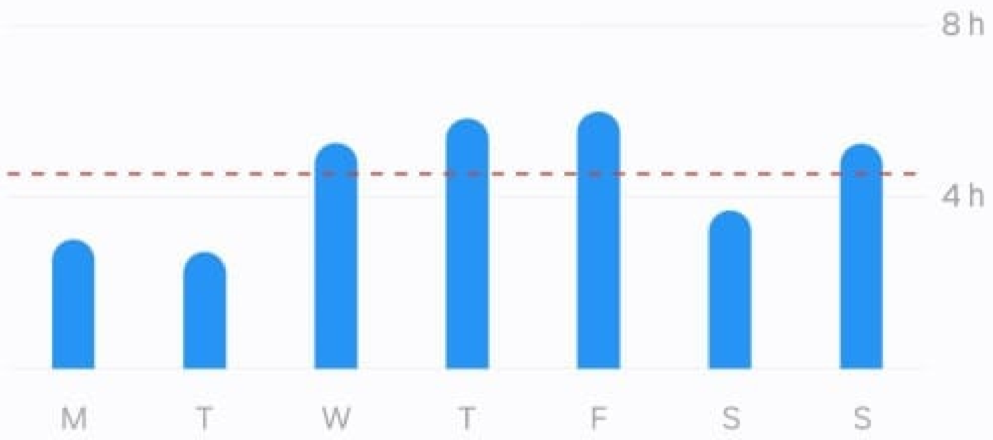

Video

5 h 16 m

52 m used on average for previous 3 weeks

YT Kids **4 h 43 m**

YouTube **33 m**

Peak usage times

31 h 51 m **Total screen time**
