# Supplementary material for: Association of excessive screen time exposure with ocular changes leading to astigmatism in children
Source: PLoS One. 2025 Apr 1;20(4):e0317961. doi: 10.1371/journal.pone.0317961 (PMC11960901; doi:10.1371/journal.pone.0317961)
Supplement: S2 Fig — (PDF) [file pone.0317961.s002.pdf]

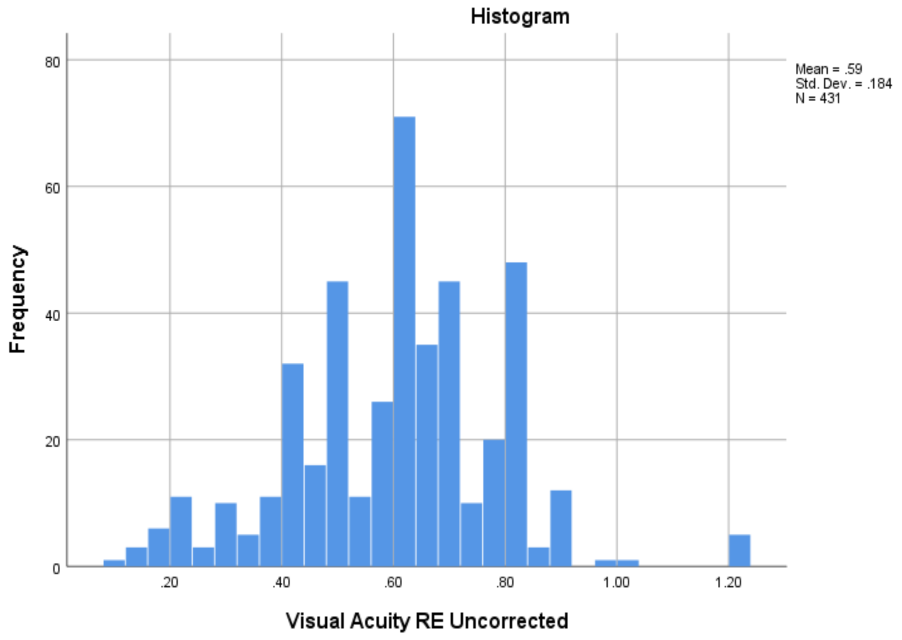
Histogram
Mean = .59
Std. Dev. = .184
N = 431
Frequency
Visual Acuity RE Uncorrected

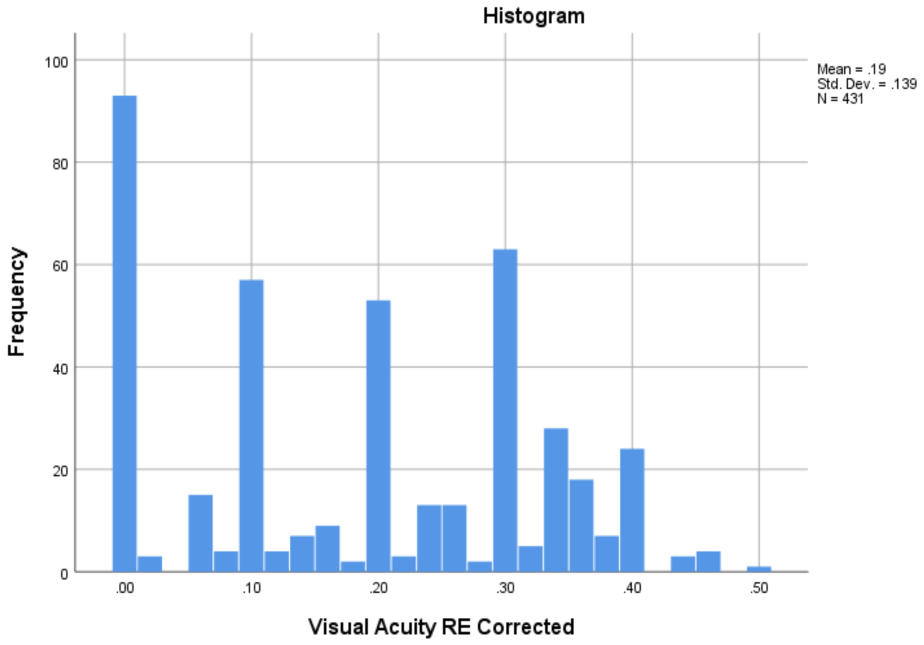
Histogram
Mean = .19
Std. Dev. = .139
N = 431
Frequency
Visual Acuity RE Corrected

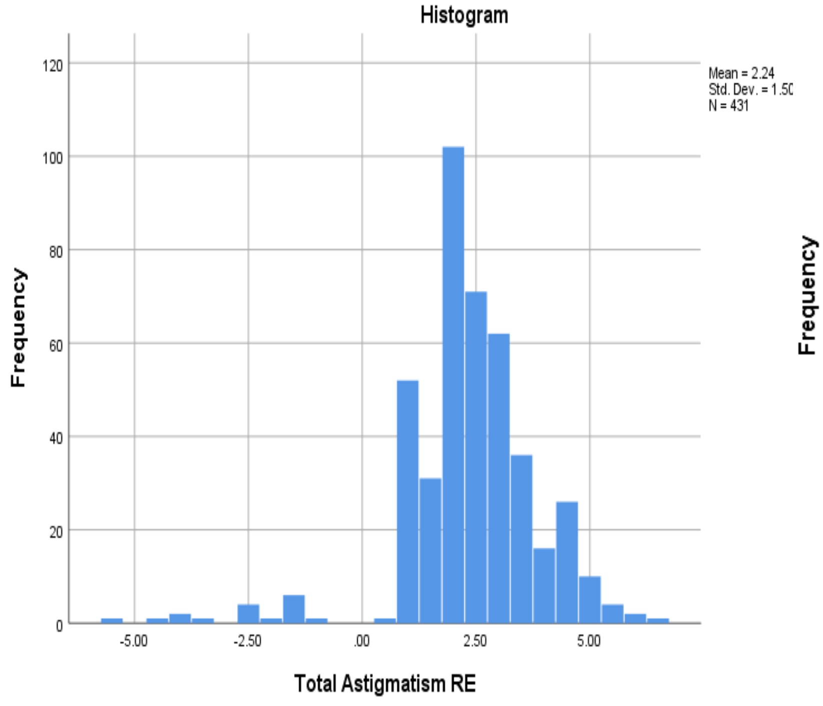
Histogram
Mean = 2.24
N = 431
Frequency
Total Astigmatism RE

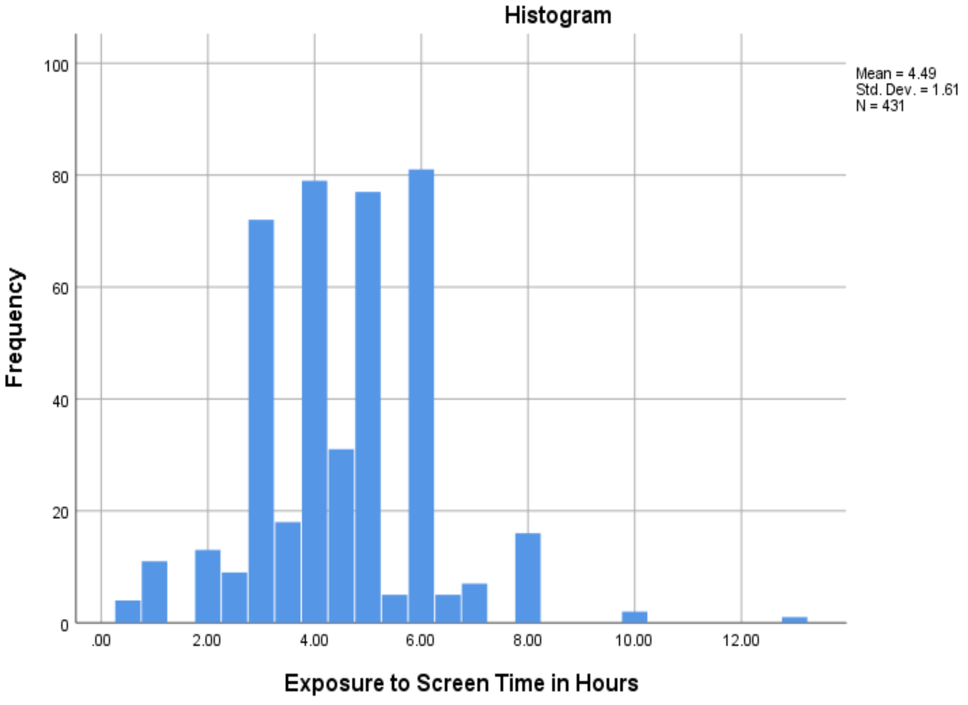
Histogram
Mean = 4.49
Std. Dev. = 1.61
N = 431
Frequency
Exposure to Screen Time in Hours

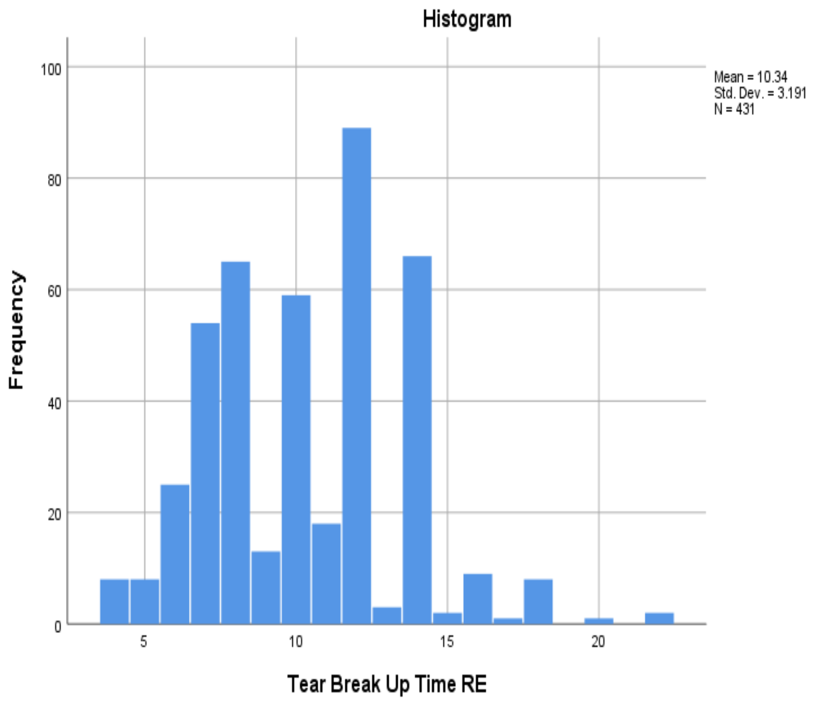

S4 Figure: The figure showed the graphical presentation of the histogram plots highlighting the normality of the data.
