# Supplementary figures and images for: Association of excessive screen time exposure with ocular changes leading to astigmatism in children
Source: PLoS One. 2025 Apr 1;20(4):e0317961. doi: 10.1371/journal.pone.0317961 (PMC11960901; doi:10.1371/journal.pone.0317961)

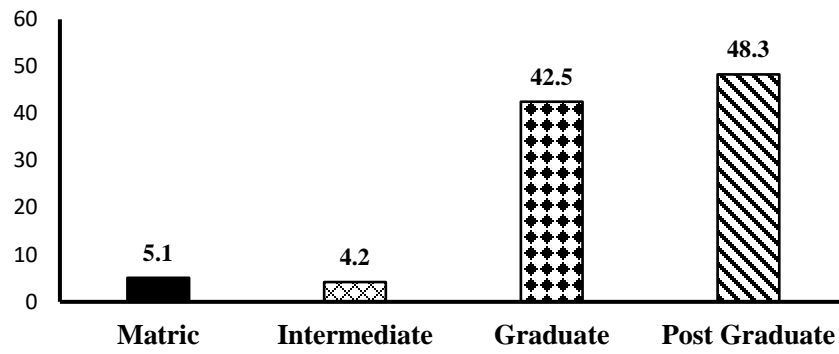

**Figure S1:** The parents of study participants were educated to graduate or post graduate level.

Supplement: S3 Fig — It showed that mostly they were educated to graduate or post graduate level. (PDF) [file pone.0317961.s003.pdf]
