# Supplementary material for: Association of excessive screen time exposure with ocular changes leading to astigmatism in children
Source: PLoS One. 2025 Apr 1;20(4):e0317961. doi: 10.1371/journal.pone.0317961 (PMC11960901; doi:10.1371/journal.pone.0317961)
Supplement: S4 Fig — (PDF) [file pone.0317961.s004.pdf]

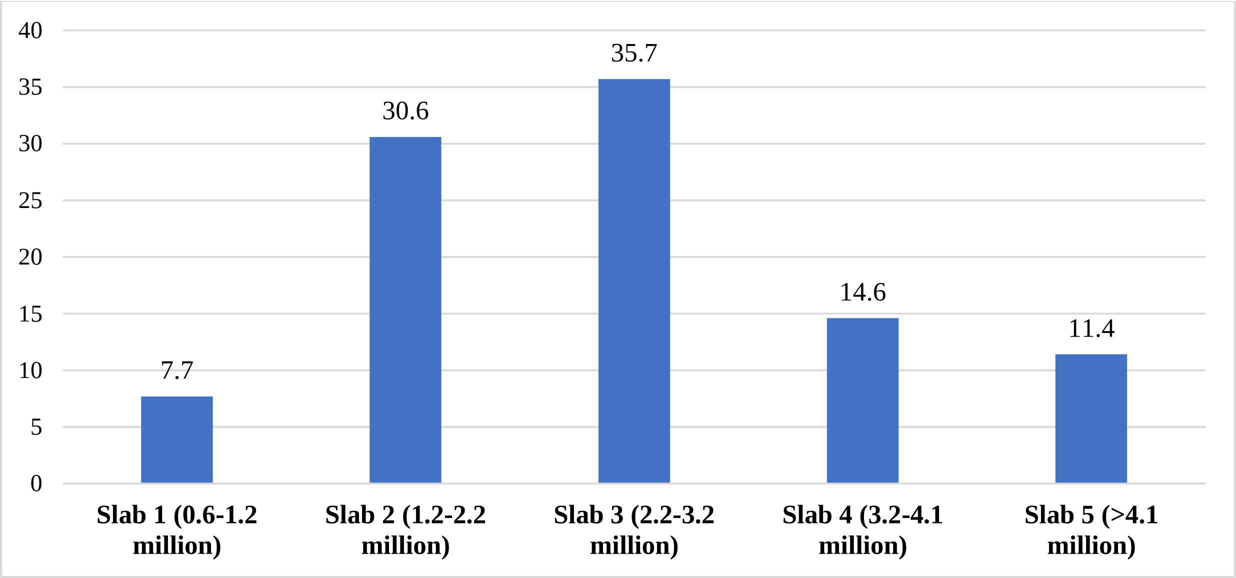

FigureS2: Presented the status of parental income status per annum as per Govt of Pakistan Tax Slab status, Majority were lying in tab slab 3 and 4.
