# Supplementary material for: Association of excessive screen time exposure with ocular changes leading to astigmatism in children
Source: PLoS One. 2025 Apr 1;20(4):e0317961. doi: 10.1371/journal.pone.0317961 (PMC11960901; doi:10.1371/journal.pone.0317961)
Supplement: S5 Fig — (PDF) [file pone.0317961.s005.pdf]

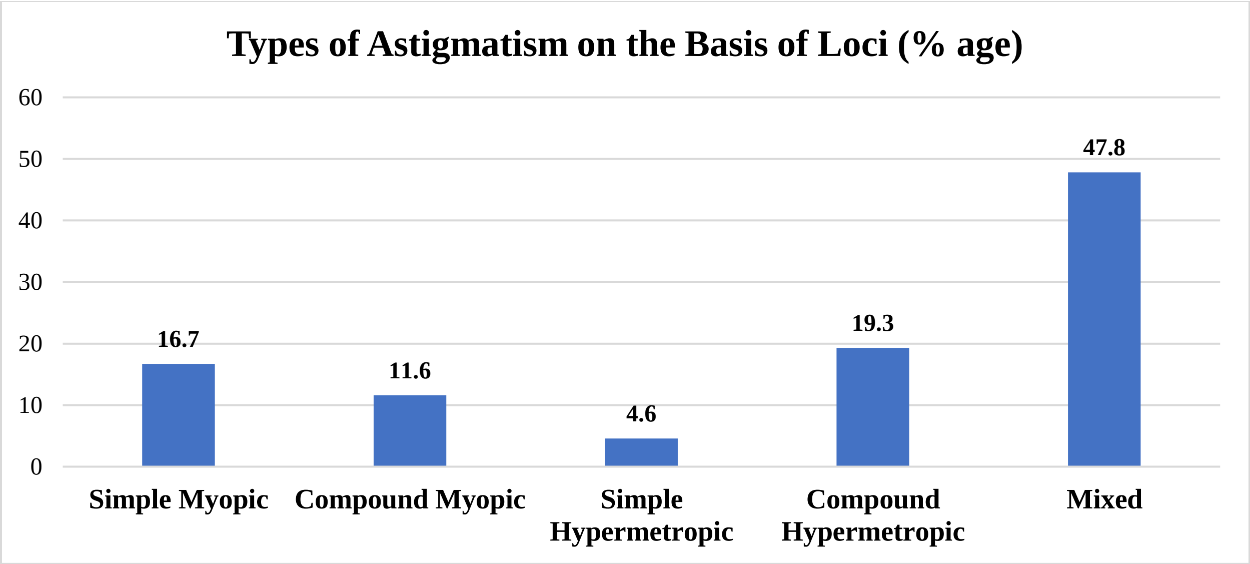

Figure S3 Represented the type of Astigmatism on the basis of Foci, it showed that majority of the study participants were diagnosed with Mixed Astigmatism followed by Myopic and Hypermetropic Astigmatism.
