# Supplementary material for: Association of excessive screen time exposure with ocular changes leading to astigmatism in children
Source: PLoS One. 2025 Apr 1;20(4):e0317961. doi: 10.1371/journal.pone.0317961 (PMC11960901; doi:10.1371/journal.pone.0317961)
Supplement: Attachment — Submitted filename: Rebuttal Letter 1.docx [file pone.0317961.s010.docx]

**Rebuttal Letter**

**Response to Editorial Office Raised Questions:**

1. **The Table 2 caption reads "The Spearman correlation analysis revealed a statistically significant negative correlation between exposure to screen time, TBUT and, total and corneal astigmatism." However, two of the 3 coefficients presented in the table are positive, not negative.**

Ans: The caption has been updated on page no 11-line no. 213-14.

2. **There appears to be a conflation between odds and risk ratios. You report that "inflammatory conjunctivitis/lid thickness have three times greater risk of developing high astigmatism (OR = 3.31, p-value < 0.001, CI = 1.91 to 5.73) while the risk of moderate astigmatism in such cases was two times higher (2.12, p-value = 0.004, CI=.26-3.56)." However, an odds ratio of 3 is not the same as a three-fold increase in risk.**

**Ans:** Thank you for raising this important distinction. You are correct that odds ratios (ORs) and relative risks (RRs) are not interchangeable, and an OR of 3 does not directly equate to a three-fold increase in risk. The odds ratio quantifies the odds of an event occurring in one group relative to another, whereas the relative risk is the ratio of probabilities. In our study, we reported odds ratios (ORs), as is typical in logistic regression analyses. The phrasing in the initial draft may have inadvertently implied a direct equivalence to relative risk, which is not accurate. We appreciate this opportunity to clarify.

While ORs and RRs can approximate each other when the event's probability is low, this relationship breaks down as event probability increases. In our case, the ORs provide a valid measure of association between inflammatory conjunctivitis/lid thickness and the likelihood of high or moderate astigmatism. To address your concern and improve clarity, we revised the response that is updated on line no 200-02 page no 10.

This approach ensures accuracy in reporting the results without conflating odds ratios with relative risks. We emphasize that the statistical results remain robust and correctly interpreted within the framework of logistic regression analysis. The association between the variables under study and astigmatism is significant and clinically meaningful.

**Reviewer 2:**

Q1. **While the authors have adequately addressed the majority of the comments, I have a concern regarding the sample size calculation. It appears that the formula used is appropriate for descriptive studies; however, given the nature of the study, a formula specific to correlational studies would be more suitable. Therefore, I request the authors to perform a post-hoc power analysis to confirm whether the sample size is sufficient to support the study's conclusions.**

Ans: We appreciate your insightful comment regarding the sample size calculation and the suggestion to perform a post-hoc power analysis to ensure the adequacy of our sample size for the study.

In response to your concern, we conducted a post-hoc power analysis using G*Power software to assess whether our sample size was sufficient to support the study's conclusions. The analysis showed that a sample size of 100 would have been adequate to achieve the desired statistical power for the study's objectives. Given that our actual sample size was 431, which is more than four times the required minimum, we are confident that our study was sufficiently powered to detect meaningful correlations and to ensure the reliability of the results.

We hope this addresses your concern, and we thank you once again for highlighting this important aspect, allowing us to further confirm the robustness of our methodology. (Fig Attached)
